# The effects of internet and social media use on the work performance of physicians and nurses at workplaces in Palestine

**DOI:** 10.1186/s12913-022-07934-2

**Published:** 2022-05-12

**Authors:** Muna Ahmead, Nida Hamamadeh, Issa abu Iram

**Affiliations:** Faculty of Public Health, Al Quds University, Jerusalem, Palestine

**Keywords:** Internet, Social application, Work performancee, Doctors, Nurses, Palestine

## Abstract

**Background:**

The use of internet and social media applications in the workplace has increased dramatically with both beneficial and harmful effects. Their use also appears to affect job performance in the workplace.

**Aim:**

To assess the beneficial and harmful effects of internet and social media application use in the workplace, and on the work performance of health professional in the major 4 referral hospitals in the cities of Hebron and Bethlehem.

**Method:**

A cross-sectional study was conducted using self-reported questionnaires: a work-related social media questionnaire (WSMQ), and the William Anderson Work Performance Scale. The sample of the study included 409 participants (169 physicians and 240 nurses).

**Results:**

The Pearson correlation test showed a weak positive statistically significant relationship between the WSMQ and William Anderson Work Performance Scale. The findings revealed that the overall mean score for the WSMQ was high (mean score 3.57), and the higher mean was for the beneficial WSMQ compared with the harmful WSMQ. The mean for the William Anderson Work Performance Scale was moderate performance (3.35). The higher mean was for Organizational Citizenship Behavior-Individual (OCBI), followed by In Role Behavior (IRB).

**Conclusion:**

Palestinian healthcare professionals should be encouraged to use internet and social media applications to improve health outcomes, and provide health information to the community rather than simply using these tools for communication. Administrative staff and policy makers in Palestinian hospitals should regulate the use of internet and social media applications in hospitals by developing a clear policy on this topic for the workplace.

## Introduction

Social media is defined as an internet-based platform that enables its users to connect, collaborate, and communicate with others in real time [1]. Social media is the most widely used platform to share information and maintain contact with people. The introduction of the internet and social media applications in the health system has increased dramatically. Househ [2] found that the use of social media among healthcare professionals is increasing. A survey of more than 4000 physicians conducted by Ferguson et al. [3] found that more than 90% of physicians use some form of social media for personal activities. Only 65% of the participants use these sites for professional reasons. Moreover, Surani et al. [4] indicated that 87.9% of healthcare professionals used social media and spent approximately one hour on social media every day. Quisenberry et al. [5] found that the use of social media among physicians and nurses was identical at 88% for each group. The time spent on social media by physicians and nurses was more than 30 min per day. In addition, 37.9% of doctors and 47.5% of nurses believed that the medical information posted on social media was correct and 61.5% of the participants advised their patients to use social media to read about their illnesses. A study by Irfan et al. [6] found high use of social media among physicians (95.4%), with 51% of them checking their accounts four times per day.

The use of internet and social media applications has a positive impact on the healthcare system. It is considered a means to share health information, discuss health issues, communicate with patients, promote primary healthcare behaviors, and can be a useful tool for collaboration. It enables the healthcare professional to hold a dialogue with their colleagues and peers, and keeps them up to date with recent healthcare developments [7]. Healthcare professionals, including physicians and nurses, can also use internet and social media applications to improve health outcomes, develop a professional network, increase awareness of new discoveries, communicate with patients during the delivery of care, and provide health information to the community [8]. Further, physicians use social media and internet to read new articles, listen to medical experts, find out about research development, and to consult colleagues regarding patient issues, in addition to personal purposes [9]. Nurses are used to delivering professional training and have influenced the nursing educational and learning experience.

Despite the benefits of social media to organizations and individuals, social media can become a threat if employees share internal and confidential information on social media applications in a manner detrimental to competitive advantage [10]. According to Wilson [11], there are five risks associated with the use of the internet and social media applications in the workplace: loss of staff productivity; leaks of information from staff into an open environment; damage to a business’s reputation; cyber crime; and loss of data due to outdated passwords. A study conducted by Erer and Çobaner [12] showed that the majority of nurses (80%) believed that the use of social media has several risks. These risks relate to inaccurate information, unprofessional behavior, violation of patient privacy and organizational risks (62, 58, 53, 33%, respectively). Nearly 26% of participants stated that there is a need for informative education or guidelines controlling the use of social media within the workplace.

One of the most important effects of the use of internet and social media applications in the workplace is the effect on work performance. Campbell [13] defined work performance as the actions or behaviors of an individual that are related to the organization’s goals. Thus, work performance focuses on the behavior or actions of an employee rather than the outcome of their behavior or actions [14]. Gaudin [15] showed that 77% of employees who use social media during working hours demonstrated a 1.5% decrease in employee productivity. This drop in productivity is due to excessive browsing and uploading of photographs unrelated to the workplace. Also, Haith [16] showed that intensive use of social media by workers may develop into internet addictive behavior which may reduce productivity in the workplace.

In Palestine, since the Israeli occupation of the Palestinian territories in 1967, Israel tightened its control over the ICT infrastructure in Palestine, inhibiting its development and prevents the establishment of an independent network to oppress and control Palestinians resulting in repeated destruction of networks and severe violations of Palestinian digital rights such as the right to privacy, access internet, freedom of opinion and expression, the right to get and disseminate information, and the right to communicate. Further, the Palestinian must connect through an Israeli company, so, Israel gets a percentage on every call from these companies to the West Bank and Gaza consumers [17].

According to the new Global Digital Review [18], the percentage of internet users in Palestine has increased to 64% with 3.25 million Palestinian users. The number of active users of social media reached 2.70 million, up by 20% from the previous year of 2019. Facebook had the largest proportion of users (72%) with 2.4 million in the West Bank and Gaza Strip.

However, there is a lack of studies to assess the beneficial and harmful effects of using internet and social media applications in the workplace and the effects on the work performance of health professional in hospitals in Palestine. This is the first study to assess the beneficial and harmful effects of internet and social media application use in the workplace, and the effects on the work performance of Palestinian health professional in the major referral hospitals of Hebron and Bethlehem.

## Methods

The aim of the study is to assess the beneficial and harmful effects of internet and social media application use in the workplace, and on the work performance of physicians and nurses in the major 4 referral hospitals in the cities of Hebron and Bethlehem. A cross-sectional design was employed using self-reported questionnaires. The target population of this study were physicians and nurses who had direct contact with patients in four referral hospitals in Hebron and Bethlehem. The hospitals were the Hebron Governmental Hospital; Al-Ahli Hospital; Beit Jala Governmental Hospital; and the Arab Society for Rehabilitation Hospital. The total population of the current study was 1307, distributed as 367 physicians and 940 nurses. The study sample was calculated using a proportional method for physicians and nurses in each hospital by computer software (PEPI-for-windows) and the sample size was found to be 297 participants (www.raosoft.com). However, 420 questionnaires were distributed by the researchers as papers and a total of 409 participants filled in the questionnaires using the convenience sampling method. The data was collected during the period from December of 2019 to January of 2020.

### Study instruments

The data collection tools used in this study were self-administered questionnaires. A socio-demographic data sheet was developed for the purpose of this study that included different questions such as age, gender, religion, place of residency, marital status, educational level, and monthly income. It also included work-related questions such as specialization, professional title, workplace, years of experience, number of working hours, and employment status. Internet-related questions included the most used internet and social media applications, frequency of internet and social media use, hours spent on internet and social media applications, devices used to access the internet, the network used to access internet and social media applications, and awareness about internet and social media application policies in hospitals.

The Work-Related Social Media Questionnaire (WSMQ) was developed by Landers and Callan [19] to assess the degree to which employees are using social media in the workplace. In the current study, the short form of the scale (17) was used; it contained two subscales of 8 beneficial WSMQ questions and 9 harmful WSMQ questions. Participants were asked to indicate their level of agreement with each statement using a 5 point Likert scale ranging from strongly disagree to strongly agree, where 1 = strongly disagree, 2 = disagree, 3 = neutral, 4 = agree and 5 = strongly agree. The beneficial WSMQ (q1-q8) assessed key items as follows: q1 assessed information gathering; q2 assessed communication with existing clients; q3 assessed new client outreach; q4 assessed crowdsourcing; q5 assessed intra-office communications; q6 assessed participation in an online work community; q7 assessed organizational reputation management; and q8 assessed social media as a technical solution.

The harmful WSMQ (q9-q17) assessed key items as follows: q9 assessed the creation of offensive content; q10 assessed time theft; q11 assessed the disparaging of others; q12 assessed multi-tasking; q13 assessed poor representation of the organization; q14 assessed diminishing personal reputation; q15 assessed the establishment of an inappropriate relationship; q16 assessed plagiarism; and q17 assessed relationship refusal, as shown in Table 4.

For the first eight questions of the questionnaire, if the participants answered agree or strongly agree, it indicated that the internet and social media applications had positive effects, while the answers for the last 9 questions of the scale agreed and strongly agreed, answers indicated negative effects.

The William Anderson Work Performance Scale was developed by William and Anderson [20]. It has 21 items to measure separate dimensions of work performance, including the performance of organizational citizenship behaviors (OCB) and employee performance of in-role behavior (IRB). The OCB has two broad categories: OCBO-behaviors that benefit the organization in general and OCBI-behaviors that benefit specific individuals and indirectly contribute to the organization [20]. The OCBI part of the scale had 7 questions (from q1-q7), OCBO also had 7 questions (from q8-q14), and IRB had 7 questions (q15-q21). The participants were asked to indicate their level of agreement with each statement using a 5 point Likert scale ranging from strongly disagree to strongly agree, where 1 = strongly disagree, 2 = disagree, 3 = neutral, 4 = agree and 5 = strongly agree, as shown in Table 5.

### Validity and reliability of scales

The Cronbach’s Alpha reliability test for overall WSMQ, WSMQ(+), WSMQ(−) and William Anderson Work Performance Scale was 0.77, 0.71, 0.80, and 0.80 respectively. The content validity of the questionnaires was examined by a committee of three experts in public health who hold doctoral degrees (PhDs) from Al-Quds University. A few changes were required by them regarding the language or content. The Work-Related Social Media Questionnaire and William Anderson Work Performance Scale were translated into Arabic language and a reverse translation was done by an English translator.

### Statistical analysis

The data were analyzed using the statistical package for social sciences (SPSS) version 23. The relationship between the socio-demographic data, the Work-Related Social Media Questionnaire, and the William Anderson Work Performance Scale was analyzed using parametric tests such as frequency, T-test, ANOVAs test, and the Pearson test.

## Results

Table 1 presents the socio-demographic characteristics. It shows that 59.1% of the participants were males and 58.5% of the participants were aged from 20 to 30 years. Regarding residency, 52.8% of the participants lived in a village and the majority of the participants (64.1%) were married or previously married. In addition, 69.0% had a bachelor’s degree and 71.1% of the participants had an income of $1600 or less.

**Table 1:** Socio-demographic variable of the participants

Variables		Percentage (N)
**Gender**	Male	59.1% (241)
	Female	40.9% (167)
**Age**	From 20–30 years	58.5% (239)
	<30–40 years	29.1% (119)
	<40-50 years	11.2% (46)
	<50 years	1.2% (5)
**Religiosity**	Islam	97.5% (398)
	Christianity	2.5% (10)
**Place of residency**	City	41.5% (162)
	Village	52.8% (206)
	Camp	5.7% (22)
**Marital status**	Single	35.9% (147)
	Married and previously married	64.1% (262)
**Educational status**	Diploma	17.8% (73)
	Bachelor	69.0% (282)
	Postgraduate	13.2% (54)
**Monthly income**	1600 $ and less	71.1% (291)
	More than 1600$	28.9% (118)

The work-related variables of the participants are presented in Table 2 and show that 58.7% of the participants were nurses and 41.3% were physicians. Around half of the participants had experience of three years and less, and around 60% worked from 8 to 16 h per day.

**Table 2:** Work related variables of the participants

Variables		Percentage (N)
**Specialization**	Medicine	41.3% (169)
	Nursing	58.7% (240)
**Workplace**	Hebron Governmental Hospital	28.3% (116)
	Al-Ahi Hospital	27.9% (114)
	Beit-Jala Hospital	23.5% (96)
	Arab Society for Rehabilitation	20.3% (83)
**Years of experience**	3 years and less	45.7% (187)
	<3–10 years	29.3% (120)
	<10 years	25.0% (102)
**Number of working hours per day**	>8 h	21.1% (86)
	8–16 h	59.1% (241)
	<16 h	19.8% (81)
**Employment status**	Part-time	12.2% (50)
	Full-time	87.8% (359)

Table 3 shows the use of internet and social media applications by physicians and nurses. The findings show that the majority of participants (64.3%) used more than two social media applications: Facebook was the most used platform (72.0%), followed by WhatsApp (13.1%). The lowest percentage of applications used was for YouTube (3.7%) and Google search platform (1.7%).

**Table 3:** Internet related variables of the participants

Variables		Percentage (N)
Internet and social media applications	One application used	17.0% (68)
	Two applications used	18.7% (75)
	More than two applications used	64.3% (258)
Most used internet and social media applications	Facebook	72.0% (291)
	WhatsApp	13.1% (53)
	Twitter	7.0% (28)
	YouTube	3.7% (15)
	Instagram	2.5% (10)
	Google	1.7% (7)
Frequency of internet and social media applications usage	Daily-most of time	71.9% (294)
	Once per day	28.1% (115)
Hours spent on internet and social media applications per day	>1 h	33.5% (137)
	From 1–3 h	45.0% (184)
	<3 h	21.5% (88)
Hours spent on internet and social media in the workplace	>1 h	76.7% (313)
	From 1–3 h	16.9% (69)
	<3 h	6.4% (26)
Hours spent on internet and social media outside the workplace	>1 h	33.5% (137)
	From 1–3 h	44.3% (181)
	<3 h	22.2% (91)
Devices used to access to internet and social media	Phone mobile	95.4% (390)
	Other devices	4.6% (19)
Reasons for using internet and social media in the workplace	Communicate colleagues	62.0% (253)
	Communicate with patients	10.3% (42)
	Skills development and Research	18.6% (76)
	Read recent news	4.9% (20)
	Entertainment. Job vacancies Chatting and Patient safety	4.2% (17)
Add patients as friends or followers	Yes	34.5% (141)
	No	65.5% (268)
The presence of policy regulates internet and social media use in hospital	Yes	47.7% (195)
	No	52.3% (214)
Does organization emphasize staff awareness about policy of internet and social media use	Yes	41.8% (170)
	No	58.2% (237)
Effect of internet and social media on work performance	Positive effect	61.5% (251)
	Not at all	22.8% (93)
	Negative	15.7% (64)

Of the participants, 71.9% reported that they used social media daily and frequently. Also, 45% reported that they spent from one to three hours per day on the internet and social media applications. Furthermore, they were asked specifically about how many hours did they spend on internet and social media applications use in workplace, and 76.7% reported that they spent less than one hour**.** In total, 95.4% of the participants reported that they used a mobile phone to access the internet and social media applications. Asked about the reasons for using internet and social media applications, 62.0% reported that it was to communicate with colleagues, and 18.6% said it was for skills development and research.

Some 65.5% of the participants stated that they did not add patients as friends or followers on the internet and social media applications. Also, more than half of the participants (52.3%) reported that their institution did not have a policy to regulate internet use. On the effect of internet and social media application use on job performance from the point of view of participants, the results ranged from an extremely positive effect to an extremely negative effect. For example, 61.5% of the participants reported that it affected their performance positively, while 22.8% answered that they it did not affect them at all, as shown in Table 3.

### Work-Related Social Media Scale results

Table 4 shows the results of the WSMQ. The overall mean of WSMQ was 3.57, which indicated that the internet and social media applications in the workplace had a strong positive effect on participants in general. When comparing the means of beneficial and harmful WSMQ among participants, a higher mean was evident (3.17), which indicates moderate beneficial effects rather than harmful WSMQ (2.08).

**Table 4:** Distribution (%) and mean of questions related to Work related Social Media questionnaire

#	Questions	Strongly Agree	Agree	Neutral	Disagree	Strongly Disagree	Mean
1	I have used social media to learn how to perform better at my job	9.8	47.2	20.8	15.2	7	3.37
2	I communicate with existing customers or clients via social media	3	31.5	17.1	28.4	20	2.68
3	I reach out to potential new customers and clients using social media	1.2	25.2	24.2	28.9	20.3	2.58
4	I request help from people on social media when I am having trouble solving a problem at work	4.9	40.8	25.2	21	8.1	3.13
5	I use social media to contact my coworkers when I am unable to reach them by other mean	20.5	51	19.3	6.8	2.4	3.80
6	I use my organizations official social media presence to network	4.4	33	30.3	19.1	13	2.96
7	If I find something on social media that will harm the reputation of my coworkers or our organization, I let people know	13.7	40.1	30.6	11.2	4.4	3.49
8	I have taken advantage of the technical features of social media to accomplish work tasks	10	39.6	30.1	14.7	5.4	3.34
9	Other people at work have offended by something I posted on social media	2.4	12	23.7	34	27.9	2.27
10	I’ve used social media when I should been working	2.4	11.7	22	35.5	28.4	2.31
11	I have discussed negative feelings towards clients on social media	1	15.9	21.3	41.8	20	2.35
12	I access social media while I am doing other work	1.2	16.4	22.5	43.5	16.4	2.42
13	I have done poor quality work using my organizations social media accounts	0.7	6.6	15.9	44.3	32.5	1.98
14	Clients have posted information about me on social media that harmed my reputation at work	0.2	5.1	12.2	40.3	42.1	1.81
15	I have invited personal relationship with a client or coworkers that I shouldn’t have	1.2	6.4	13.7	37.2	41.5	1.88
16	I’ve stolen information or other content from social media and used it as if it was my own work	0.5	6.8	11.2	34.5	47	1.79
17	I’ve created an uncomfortable situation by refusing connections with coworkers, supervisors, or customers via social media	1.2	6.8	14.9	37	40.1	1.92
Overall Work related Social Media Questionnaire							3.57
Beneficial Work related Social Media Questionnaire							3.17
Harmful Work related Social Media Questionnaire							2.08

Just three questions showed that the internet and social media applications affected most participants positively and 50% or more of the participants answered agree or strongly agree. For example, the highest means and percentages (>50% of strongly agree and agree) were for the questions, “I use social media to contact my coworkers when I am unable to reach them by other means” (71.5%, M = 3.80), and “I have used social media to learn how to perform better in my job” (57%, M = 3.37). For harmful WSMQ, the mean was 2.08, which indicated low harmful effects of the internet and social media applications in the workplace.

### The relationship between the beneficial and harmful WSMQ and specialization

The findings showed that there was a statistically significant relationship between beneficial WSMQ and specialization (*p*-value = 0.000). For example, physicians had higher beneficial social media usage than nurses (see Table 5).

**Table 5:** The relationship between the beneficial and harmful WSMQ and specialization

Independent variable		Beneficial WSMQ			Harmful WSMQ		
		Mean	T/F	*P*-value	Mean	T/F	*P*-value
Specialization	Medicine	3.0303	3.881	0.000	3.9967	1.636	0.103
	Nursing	2.8103			3.8875		

In addition, The Pearson correlation test showed a negative statistically significant between the beneficial and harmful WSMQ (*r* = −0.158؛ *p* = 0.001) as seen in Table 6.

**Table 6:** Pearson correlation between the beneficial and harmful WSMQ

	Harmful WSMQ		
**Beneficial WSMQ**	**R**	**Sig**	**N**
	**−0.158**	**0.001**	**409**

As seen in Table 7, the results showed that the mean of the William Anderson Work Performance Scale was 3.35, which indicated moderate work performance among participants. In the three subscales, the highest mean was for organizational citizenship behaviors-individuals (OCBI) (3.68), followed by in-role behaviors (IRB) with a mean of 3.24. The lowest mean (3.15) was for organizational citizenship behaviors-organizations (OCBO). There was a high performance for OCBI and moderate for IRB and OCBO among the participants.

**Table 7:** Distribution (%) and mean of questions related to William Anderson Work Performance Scale

#	Questions	Strongly Agree %	Agree %	Neutral %	Disagree %	Strongly Disagree %	Mean
1	I help others who have been absent	17.8	63.6	11.7	4.2	2.7	3.89
2	I help others who have heavy workloads	20.8	60.1	13.4	4.3	1.2	3.95
3	I assist my supervisor with his/her work	10.8	50.4	27.9	7.8	2.9	3.58
4	I take time to listen to co-workers’ problems and worries	7.6	46.9	32.3	9.8	2.9	3.46
5	I go out of my way to help new employees	14.0	50.1	29.3	4.2	2.4	3.68
6	I take a personal interest in other employees	12.5	51.1	26.8	7.6	2.0	3.64
7	I pass along information to co-workers	12.2	48.2	25.9	8.6	5.1	3.53
8	My attendance at work is above the norm	9.3	40.3	37.0	10.5	2.2	3.44
9	I give advance notice when unable to come to work	24.4	52.3	16.2	5.1	1.5	3.93
10	I conserve and protect organizational property	28.1	48.9	18.1	3.4	1.5	3.98
11	I adhere to informal rules devised to maintain order	17.4	51.5	24.0	4.4	2.2	3.77
12	I adequately complete my assigned duties	22.5	53.1	20.0	3.4	1.0	3.92
13	I fulfill the responsibilities specified in my job description	22.7	55.5	15.9	4.2	1.5	3.94
14	I perform the tasks expected of me	21.3	56.7	16.6	4.7	0.7	3.93
15	I meet the formal performance requirements of the job	17.1	48.8	25.4	7.3	1.2	3.73
16	I engage in activities that will directly affect my performance	10.8	36.7	32.3	15.6	4.4	3.33
17	I take undeserved work breaks*	3.7	13.7	26.6	36.2	19.6	2.45
18	A great deal of my timk2e is spent on personal phone/email –communications	3.2	8.6	21.0	42.1	24.9	2.22
19	I complain about insignificant things at work	2.7	9.3	21.8	44.4	21.8	2.26
20	I neglect aspects of my job that I am obligated to perform	2.2	5.1	11.0	44.7	37	1.90
21	I fail to perform essential duties	1.5	6.6	12.7	39.6	39.6	1.91
William Anderson Work Performance scale							3.35
Organizational citizenship behaviors- Individual (OCBI)							3.68
Organizational citizenship behaviors-Organization (OCBO)							3.15
In- role behaviors (IRB)							3.24

The results also showed that most of the participants reported that their work performance was moderate or good; more than 50% of the participants answered strongly agree and agree for 14 out of 21 questions. For example, 81.4% answered, “I help others who have been absent”, followed by, “I help others who have heavy workloads” (80.9%), “I fulfill the responsibilities specified in my job description” (78.2%), and “I perform the tasks expected of me” (78.0%).

The lowest percentages (<50% for strongly agree and agree answers) were for seven questions. For example, 49.6% answered, “My attendance at work is above the norm”, followed by, “I engage in activities that will directly affect my performance”(47.5%), and “I take undeserved work breaks” (17.4%).

The Pearson correlation (r) test was used to test the correlation between the Work-Related Social Media Questionnaire and Work Performance Scale, as seen in Table 8 The Pearson correlation test showed a weak positive statistically significant relationship between the Work-Related Social Media Questionnaire and William Anderson Work Performance Scale (*r* = 0.198؛ *p* = .000). When testing this correlation with the three subscales of the William Anderson Work Performance Scale, a statistically significant positive relationship was found in the OCBI subscale (*r* = 0.381؛ *p* = 0.000), but there was no correlation between the other subscales OCBO (*r* = −0.003؛ *p* = 0.956) and IRB (*r* = 0.032؛ *p* = 0.513) (see Table 8).

**Table 8:** Pearson correlation (r) between the Work related Social Media Questionnaire and William Anderson Work Performance Scale

	William Anderson Work Performance Scale											
	Overall			OCBI			OCBO			IRB		
**WSMQ**	R	Sig	N	R	Sig	N	R	Sig	N	R	Sig	N
	0.198	.000*	409	0.381	.000*	409	−0.003	.956	409	0.032	.513	409

*P value = *p* < 0.05

### The relationship between Work related Social Media Questionnaire and William Anderson Work Performance Scale and other variables

The findings in Table 9 showed that there was a statistically significant relationship between the Work related Social Media Questionnaire and monthly income and specialization.

**Table 9:** The relationship between the Work related Social Media Questionnaire and Work Performance scale and the socio-demographic variables

Independent variables		WSMQ scale			Work Performance scale		
		Mean	T/F	***P***-value	Mean	Test	***P***-value
**Gender**	Male	3.55	−1.22	**.**222	3.39	2.11	.035*
	Female	3.60			3.30		
**Age group**	20-30 years	3.55	0.81	.485	3.32	1.76	.154
	>30-40 years	3.60			3.41		
	>40-50 years	3.62			3.40		
	>50 years	3.43			3.36		
**Place of residency**	City	3.58	0.79	.454	3.36	0.29	.748
	Village	3.55			3.37		
	Camp	3.47			3.30		
**Marital status**	Single	3.55	−0.53	.596	3.30	−2.11	.035*
	Married and previously married	3.58			3.39		
**Educational level**	Diploma	3.57	2.30	.101	3.32	1.42	.243
	Bachelor	3.55			3.35		
	Postgraduate	3.68			3.44		
**Specialization**	Medicine	3.68	4.49	.000*	3.35	−0.13	.891
	Nursing	3.49			3.36		
**Monthly income**	1600 $and less	3.53	−2.68	.008*	3.33	−2.24	.025*
	> 1600$	3.66			3.42		

*P value = *p* < 0.05

Also, there were statistically significant relationships between work performance and gender, marital status and monthly income.

## Discussion

The findings showed that the majority of participants used more than two internet and social media applications. Facebook was the most used platform, followed by WhatsApp. The platforms with the lowest use were Twitter, YouTube, and Google. Facebook is favored by healthcare professionals due to its simplicity, availability of a wide range of features, and an easier way to connect with friends and families [21]. Similarly, Loeb et al. [22] showed that most physicians and nurses in hospitals were Facebook users (93.0%), followed by Twitter (36.0%). A study by Adilman et al. [23] found that Facebook was the most used platform among physicians (86.0%). The high use of facebook among the participants in this study may be because Facebook provides people with a space for the recognition of the socio-political and cultural identity which may affect their emotion postivelly. Also, due to Iseali political restriction, checkpoiunts, and Walls which separate Palestinians from their places of work, and families and make travel and communication difficult, Facebook may facilitate their connrction with otheras in order to share their daily events such as death, marriage, and education [17] m. However, Palestinian healthcare professionals should be encouraged to use social media for purposes other than Facebook communication, such as Google, to improve health outcomes, develop professional networks, keep abreast of new discoveries, and provide health information to the community.

The current study showed that 95.4% of the participants used their own mobile phone to access the internet and social media applications. These findings were supported by the study of Ozdalga et al. [24] which found that mobile phones are mostly used by physicians because they are flexible to use for communication, short text messages, sharing e-mail, web searching, high-quality cameras, and sound recorders. Inconsistently, the Fuoco and Leveridge [25] study found that laptops were the main device (55.2%) used to access the internet and social media applications by Canadian urologist participants.

The study findings revealed that the majority of participants used the internet and social media applications daily and most of the day, while 28.1% said they used it once per day. These results were supported by the Erer and Cabner [12] study which showed that 65.8% of the participants used the internet and social media applications every day. For the use of internet and social media applications in the workplace, the majority of participants (76.7%) reported spending less than one hour. Consistently, the Surani et al. [4] study found that the majority of healthcare providers (67.4%) spent less than one hour on internet and social media applications in the workplace. This finding can be explained by the fact that physicians and nurses in hospitals have a heavy workload and do not have enough time for using internet and social media applications. Many physicians who engage in social media are doing so at the expense of their other commitments such as patient care, particularly in the biggest referral hospitals (Evariant 2019; [26, 27]).

Asked for the reasons for using the internet and social media applications in the workplace, 62.0% of participants reported that they used these to communicate with their colleagues, followed by 18.6% for skills development and research, and 10.3% to communicate with their patients. The findings of the current study were supported by the Long et al. [28] study which found that 66.7% of Chinese urologists used social media for collaboration with peers, and 52.7% for surgical and medical education. Similarly, one study indicated that 93.4% of Spanish physicians and nurses used internet and social media applications for pharmacological consultation and professional communications with colleagues [29]. The Palestinian Digital Activism Report [30] found that the most common reason (75%) for using internet and social media applications among Palestinians was to communicate with friends and colleagues. Von-Muhlen and Ohno-Machado [31] found that social media could be used as a powerful communication system for healthcare workers through which they can find patient health information and past medical history. Another strong reason for using social media applications for communication in Palestinian hospitals may be because it is cheap in comparison with the expensive costs of mobile and telephone calls because the Palestinian must connect through an Israeli cellular company. So, Israel gets a percentage on every call to the West Bank and Gaza. In addition Palestinian cellular.

networks do not cover all areas in the West Bank, which forces these networks to roam on Israeli carrier networks. This means that increasing the cost of mobile calls for the Palestinians [17].

The majority of participants (65.5%) reported that they did not add their patients as friends or followers on internet and social media applications, although 34.5% of participnts responded that they did. Physicians or nurses should not add patients as friends on social media because the relationship must be terminated once the patient is discharged from hospital. Langenfield et al. [32] found that when a patient becomes a friend with nurses on social media, the nurse is exposed to a highly risky situation and their professional image may be affected. Similarly, Duymus et al. [33] found that 94.2% of Turkish physicians did not accept friend requests from patients because it is unethical and detrimental to the patient-physician relationship. Palestinian nurses and doctors need to be made aware of the dangers of adding their patients as friends or followers on internet and social media applications. Another reason that may prevent healrth professionals in Palestine from adding others to their social media application is the violation of privacy because when Palestinians use social media to express their opinions about Israeli occupation of their land on facebook,. hundreds of Palestinians were arrested by the Israeli Authorities [17].

More than half of the participants (52.3%) reported that their institution did not have a policy for internet regulation. Backman et al. [34] found that 54.0% of physicians reported that their institutions had an internet and social media regulation policy. However, Surani et al. [4] found that 40.8% of healthcare providers had an internet and social media regulatory policy. Healthcare organizations need a formal and clear policy to regulate social media applications to prevent complications and professional problems [35]. Therefore, Palestinian hospitals should have a clear policy for internet and social media application use in the workplace to prevent professional problems. However, one of the challenges for developing a policy for internet regulation in Palestine is that Isreal controls the ICT infrastructure and Palestinian must connect through an Israeli company. In addition, many Palestinias prefer to buy Israeli Internet chip because is cheaper in comparison with Palestinian internet network in West Bank. These challenges makes it difficult for the Palestinian health institutons to contrl internrt use inside them [17].

Some 58.2% of participants stated that they had not been informed of any institutional policy. Wang et al. [36] indicated that 52.7% of nurses reported that their institution did have a policy for internet and social media application use but there were no formal or unified guidelines. It is crucial to understand the policy of an institution in order to protect confidential medical information and patient privacy. Finally, when asked about the effect of using internet and social media applications on job performance from the participants’ point of view, the findings showed that the majority of participants (61.5%) reported a positive effect on their performance. These findings were congruent with the study of Long et al. [28] which found that 75.9% of Chinese urologists reported that internet and social media applications affected their performance positively because social media improved the effectiveness of medical education, increased exposure of their practices, and was a source of positive feedback from patients. Similarly, Wang et al. [36] found that 84% of nurses believed that social media applications affected their work performance in a positive manner.

### Work-Related Social Media Questionnaire and William Anderson Work Performance Scale results

The results of the study revealed highly positive effects of internet and social media application use in the workplace, as reported by the participants. The mean of WSMQ was 3.57, which indicated that the internet and social media applications in the workplace had highly positive effects in worplace. The mean of the beneficial was 3.17, which was higher than the harmful WSMQ (2.08). However, the total mean of the William Anderson Work Performance Scale was 3.35, which indicated that work performance of the participants was moderate. The Pearson correlation test showed a weak positive statistically significant relationship between the Work-Related Social Media Questionnaire and William Anderson Work Performance Scale (*r* = 0.198؛ *p* = .000). One possible explanation for this discrepancy is the use of the self-reported questionnaire. Demetriou et al. [37] indicated that one of the main disadvantages of using a self-reported questionnaire with healthcare workers is the possibility that the participants provide invalid answers or do not answer the questions truthfully and carefully for subconscious reasons. These results were supported by Gaudin [15] who found that 77% of employees who use social media during working hours resulted in a 1.5% decrease in employee productivity due to excessive browsing and the uploading of photos unrelated to the workplace. It was found that the intensive use of social media in the workplace has a substantial impact on the employees’ performance by enhancing job satisfaction, which leads to organizational commitment. However, moderate use of social media creates a balance between the personal and the professional issues of an employee because social media use increases overall performance by helping employees to improve their skills, knowledge, productivity, and enhances communication [38]. Therefore, regulations regarding the use of internet and social media applications in Palestinian hospitals should be drawn up by administrative staff and policy makers.

For the beneficial WSMQ, the highest percentages (>50% of strongly agree and agree) were for intra-office communication, followed by information gathering and organizational reputational management. The findings of the current study were consistent with a study conducted by Alshakhs and Alanzi [39] which found that social media in the workplace was a beneficial tool that improved healthcare services and enhanced medical information. Similarly, Hazzam and Larech [40] revealed that the main benefit of social media for healthcare professionals was for updating medical information and interpersonal communications. The findings of the current study were inconsistent with the study by Erer and Cobaner [12] who found that the majority of nurses (80%) believed that social media applications had negative effects such as inaccurate information, unprofessional behavior, and violation of patient privacy. The high percentage of positive responses in the current study may be because the participants exaggerated their answers, or maybe these social applications improve the psychological status of health professionals working under pressure by enabling them to communicate with others.

The findings of the current study revealed that the total mean of the William Anderson Work Performance Scale was 3.35, which indicated moderate work performance by participants. When the means of the three classes of employee behaviors were measured on the William Anderson Work Performance Scale, it showed that the highest mean was for organizational citizenship behaviors-individuals (OCBI), followed by in-role behaviors (IRB), while the lowest mean was for organizational citizenship behaviors-organizations (OCBO). A high performance for OCBI and moderate for IRB and OCBO among participants means that OCBI, such as helping other workers, had a greater effect on work performance than OCBO, such as the instructions and orders of the organization. These results were supported by the Yuxiu et al. [41] study which found that the job performance level among nurses was moderate. It is similar when employees feel that they are treated fairly by a company, have a good relationship with their manager, and have a manager who is supportive and rewards high performance. When employees are treated well, they reward the company by performing their job more effectively [42]. On other hand, the results of the current study were lower than those in other studies. For example, Qtait and Sayej [43] found that the level of job performance among nurses in five Hebron hospitals was high (71%). Mokhtar and Mohamed [44] also found that the mean job performance among professional nurses in pediatric and intensive care units was high (3.82).

Finally, the higher means of internet and social media applications were seen in a high monthly income group and physicians. It was found that people with higher incomes are more likely to use social media than people with lower incomes [45]. Similarly, Ventola [7] found that physicians were higher users of social media than other healthcare professionals because they listen to experts, research for medical developments, consult colleagues regarding patient issues and discuss practice management challenges. Higher work performance was seen in males, married participants, and high monthly income group. These results were similar to other studies [46, 47]. This might be due to work home conflict as females had a lot of demands between their families and home responsibility and their work. Also, Crawly and Grant [48] found that married employees are less absent due to economic pressures and family requirements.

### Limitations

There are some limitations in this study. The study utilized cross sectional design, this type of design may have limitations in the generalization of the results to a wider population since it measures both the prevalence of the outcomes and the determinants in a population at a point in time or over a short period of time. Also, the data collection for this study was done by using a self- administered questionnaires. So, the reliability of the results may be affected, since the participants may hesitate to express their points of view or they may describe their own thoughts, feelings or behaviors in the spurious way to please the researcher. Finally the sample included physicians and nurses who work in 4 referal hospitals which may limit the generalization of the findings to other healthcare professionals or other hospitals.

## Conclusion

The main finding of the study revealed that there was a weak positive statistically significant relationship between the Work related Social Media Questionnaire and William Anderson Work Performance Scale. Therefore, Palestinian healthcare professionals should be encouraged to use internet and social media applications to improve health outcomes, and develop a professional network. Also, administrative staff and policy makers in Palestinian Minstry of Health and hospitals should regulate the use of internet and social media applications in hospitals by developing a clear policy on this topic for the workplace.

## Data Availability

Materials for this study are available by emailing the corresponding author. The data are not publicly available due to ethical restrictions because this study is conducted in a governmental hospitals which prevents sharing data as it could compromise the privacy of research participa.
